# Simulation of melanoblast displacements reveals new features of developmental migration

**DOI:** 10.1242/dev.160200

**Published:** 2018-06-11

**Authors:** Pascal Laurent-Gengoux, Valérie Petit, Zackie Aktary, Stuart Gallagher, Luke Tweedy, Laura Machesky, Lionel Larue

**Affiliations:** 1 Laboratory Mathematics in Interaction with Computer Science (MICS), Centrale Supélec, Université Paris Saclay, Gif-sur-Yvette 91190, France; 2 Institut Curie, PSL Research University, INSERM U1021, Normal and Pathological Development of Melanocytes, Orsay 91405, France; 3 Univ Paris-Sud, Univ Paris-Saclay, CNRS UMR 3347, Orsay 91405, France; 4 Equipe Labellisée Ligue Contre le Cancer, Orsay 91405, France; 5 CRUK Beatson Institute, University of Glasgow, Garscube Estate, Switchback Road, Bearsden, Glasgow G61 1BD, UK

**Keywords:** Migration, Trajectories, Mouse, Memory

## Abstract

To distribute and establish the melanocyte lineage throughout the skin and other developing organs, melanoblasts undergo several rounds of proliferation, accompanied by migration through complex environments and differentiation. Melanoblast migration requires interaction with extracellular matrix of the epidermal basement membrane and with surrounding keratinocytes in the developing skin. Migration has been characterized by measuring speed, trajectory and directionality of movement, but there are many unanswered questions about what motivates and defines melanoblast migration. Here, we have established a general mathematical model to simulate the movement of melanoblasts in the epidermis based on biological data, assumptions and hypotheses. Comparisons between experimental data and computer simulations reinforce some biological assumptions, and suggest new ideas for how melanoblasts and keratinocytes might influence each other during development. For example, it appears that melanoblasts instruct each other to allow a homogeneous distribution in the tissue and that keratinocytes may attract melanoblasts until one is stably attached to them. Our model reveals new features of how melanoblasts move and, in particular, suggest that melanoblasts leave a repulsive trail behind them as they move through the skin.

## Abstract (precis)

**Summary:** A mathematical model to simulate melanoblast distribution in the embryonic mouse epidermis, based on keratinocyte attraction, melanoblast repulsion and melanoblast memory of their previous path.

## INTRODUCTION

Cell migration is a fundamental effector of cell positioning during embryogenesis, homeostasis and in pathogenesis. Melanoblasts (Mbs), emerging from the neural crest, have the capacity to migrate from the roof plate of the neural tube to the ventral part of the embryo through the matrix, the dermis and finally the epidermis (Petit and Larue, 2016), which is mainly composed of keratinocytes (Kcs). Melanoblasts are first uniformly dispersed in the epidermis prior to being concentrated in the hair follicles. During the migration of melanoblasts, development of the skin and growth of the embryo, various cellular mechanisms occur, including proliferation. Around birth, melanoblasts differentiate into melanocytes; they produce melanin and transfer this pigment to keratinocytes to generate coat color and skin pigmentation.

Melanoblasts migrate actively on the basement membrane that separates the dermis from the epidermis (Luciani et al., 2011) using classical fundamental molecular mechanisms involved in migration and stimulation (Mort et al., 2015; Petit and Larue, 2016). Proteins of the migration machinery include the tubulin network, actin filaments and associated proteins such as Rac1, Arp2/3 and Scar/Wave, and the transmembrane integrin proteins (Krause and Gautreau, 2014). Integrins have a dual function in migration; they have structural and signaling roles. A dialog between the different cells in the epidermis [homotypic, (Kc-Kc); heterotypic, (Mb-Kc)] is necessary to allow a functional harmony and, maybe more importantly, to ensure similar and consistent development between different individuals. In melanoblasts, proteins located at the membrane sense environmental signals to proliferate and migrate. These proteins include RTKs (receptor tyrosine kinases) such as Kit, which interacts with soluble and insoluble Steel (also known as Scf and Kitl) produced by keratinocytes, and GPCRs (G-protein-coupled receptors). Another major receptor is Ednrb (endothelin receptor B), which interacts with endothelin ligands (Edn1 and Edn3) also produced by keratinocytes. Modifications of one of these proteins in melanoblasts (cell-autonomous) or in keratinocytes (cell non-autonomous) may affect melanoblast migration, as has been shown for Kit/Steel, Ednrb/Edn3, Rac1, Cdc42, Prex1 and fascin (Lamoreux et al., 2010; Li et al., 2011; Lindsay et al., 2011; Ma et al., 2013; Silvers, 1979; Woodham et al., 2017). The physical interaction of melanoblasts with keratinocytes through cell-cell adhesion proteins such as E-cadherin is also important for cell-cell segregation and the dynamic cohesion of tissues (Hsu et al., 2000; Pla et al., 2001).

During development, melanoblasts are homogenously distributed within the epidermis until their specific localization to the hair follicle (Luciani et al., 2011; Mort et al., 2016). In order to have a homogenous dispersion of melanoblasts in the epidermis, equilibrium between attraction and repulsion may occur. Attraction could be driven by chemotactic molecules such as Steel. Repulsion may occur either through ligands and receptors (e.g. Robo-Slit) or through direct cell-cell contact. Four Robo receptors (Robo1 to Robo4) and three Slit ligands (Slit1 to Slit3) are present in melanocyte and melanoma cells (Rambow et al., 2015), whereas only Robo1, Robo3 and Slit1 are present in E15.5 mouse melanoblasts (Colombo et al., 2012). The presence of these receptors and ligands suggests the possibility that melanoblasts may instruct each other during migration. Although it has never been shown for melanoblasts, contact inhibition of locomotion (CIL) occurs through direct cell-cell contact in *Xenopus* neural crest cells, via their protrusions. Contact between protrusions in neural crest cells of *Xenopus* embryos results in inhibition of cell protrusions and resetting of intracellular polarity (Carmona-Fontaine et al., 2008; Mayor and Carmona-Fontaine, 2010). However, it has recently been described that chick neural crest cells do not use CIL to guide migration (Genuth et al., 2018). An alternative to CIL is chemical repulsion or depletion of chemoattractant substances in the skin.

To better understand the migration of melanoblasts among the basal keratinocytes of the epidermis, *ex vivo* migration of melanoblasts can be followed using genetically engineered mice (Mort et al., 2010). A mouse line expressing a melanoblast-specific Cre-recombinase (Tyr::Cre) crossed with Z/EG, Tomato or Rosa26-YFP reporter mouse lines will generate fluorescent melanoblasts that can be visualized by live-imaging microscopy (Delmas et al., 2003; Muzumdar et al., 2007; Novak et al., 2000; Srinivas et al., 2001). This approach has shed light on the mechanisms associated with the migration of melanoblasts using appropriate mouse mutants such as Rac1, Cdc42, Prex1 and fascin (Li et al., 2011; Lindsay et al., 2011; Ma et al., 2013; Woodham et al., 2017).

Here, we present a mathematical model, based on biological data, assumptions and hypotheses, in order to simulate the migration of wild-type melanoblasts in the epidermis. Once the simulation was performed, independent experimental sets of melanoblast trajectories were used to contest it. Finally, this model was challenged with the migration of melanoblasts mutated for Rac1.

## RESULTS

**Fig. 1.**
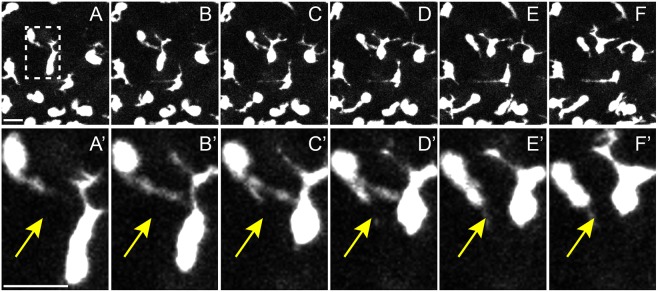
**Cell-cell contact between migrating Mbs may result in retraction of contacting pseudopods.** (A-F) Confocal image frames taken every 5 min from live *ex vivo* E15.5 wild-type Z/EGFP mouse embryo skin. Dashed box highlights contact between two Mbs. (A′-F′) Enlarged images from A-F; yellow arrows highlight protrusion and retraction of the contacting pseudopod. Scale bars: 10 μm.

The mathematical model was built on data from the published literature, assumptions and hypotheses. According to the literature, basal keratinocytes are in contact with the epidermal basement membrane, organized as a plane tissue and have a polygonal shape (Nordlund et al., 1998). Melanoblasts are also in contact with the basement membrane and migrate between the keratinocytes with the help of their protrusions. Melanoblast densities in the epidermis within basal keratinocytes are known for specific embryonic stages. Most melanoblast trajectories define plane curves (Li et al., 2011; Lindsay et al., 2011; Luciani et al., 2011; Ma et al., 2013; Mort et al., 2016; Woodham et al., 2017). The two main biological assumptions are based on general knowledge and observations of *in situ* movement of melanoblasts during embryonic development. First, keratinocytes attract distant melanoblasts by a gradient of soluble factors until contact with a melanoblast. Upon Kc-Mb cell-cell contact, ‘bound’ keratinocytes stop attracting melanoblasts. Second, two melanoblasts instruct each other through various processes, including via cell-cell contact of protrusions or cell bodies, as they cannot occupy the same physical space (Fig. 1). Overall, we named this observation ‘repulsion’.

Usual analyses for cell displacements characterizes random properties of displacements: statistical dispersion of instantaneous velocities, correlation between velocities at successive times or/and direction persistence in order to explain straight movements and direction changes. These analyses are consistent with the idea that a cell is moving on its own with a lot of independent interactions. Our aim is to explore the hypothesis that melanoblast displacements are a consequence of global interactions between melanoblasts and keratinocytes, which is not in contradiction with the fact that local or instantaneous properties of melanoblast displacements seem random. It is therefore of interest in a global perspective to study global geometric properties of melanoblast trajectories. That is why we use a method of analysis of curves based on a mathematical tool close to principal component analysis (PCA) allowing the comparison of the curve regularity and geometric properties such as turning points or loops. Ultimately, we realize that both methods independently analyze the cell trajectories per se and the crosses between trajectories.

### The mathematical model

#### Outline of the mathematical model

We have built a very general model for two populations of cell types, keratinocytes (Kcs) and melanoblasts (Mbs), assuming that Kcs were not dramatically moving during a 3 h time period and that Mbs were migrating amid Kcs. The model has many features in common with lattice gas automata (D'humières and Lallemand, 1986) and can be considered as a spatial prey-predator model with migration (Molina et al., 2015; Sadovsky and Senashova, 2016). However, it does not assess the link between cell morphology and forces (Aubry et al., 2015); therefore, Mb velocities cannot be extracted and are instead provided as experimental data. The model is based on a geometric representation of Kc and Mb, a time discretization for calculating the Mb displacements and, at each time step, a calculation of the Mb displacements depending on the Mb-Mb and Mb-Kc interactions. The model allows us to test numerous hypotheses about the causes of displacement. The general mathematical model can be summarized in three parts. In the first part, named the geometrical model, Kcs are modeled as polygons in a square domain. The polygon vertices and edges define the nodes and edges of a graph. Mbs are then randomly positioned on the nodes to initiate the process. The second part, the dynamic steps, corresponding to Mbs moving, is the updating of Mb positions and Kc characteristics at each time period. Third, Mb trajectories are analyzed.

#### The geometrical model

To build a first and simple geometric model, we limited ourselves to a plane representation, which represents the layer of cells in direct contact with the basement membrane. We considered that the domain of study is a square that makes it compatible with the experimental movie pictures (see Movies S1-S9). As we aim to model the characteristics of Kcs, and interactions between Mbs and Kcs, we have to represent both Kcs and Mbs.

**Fig. 2.**
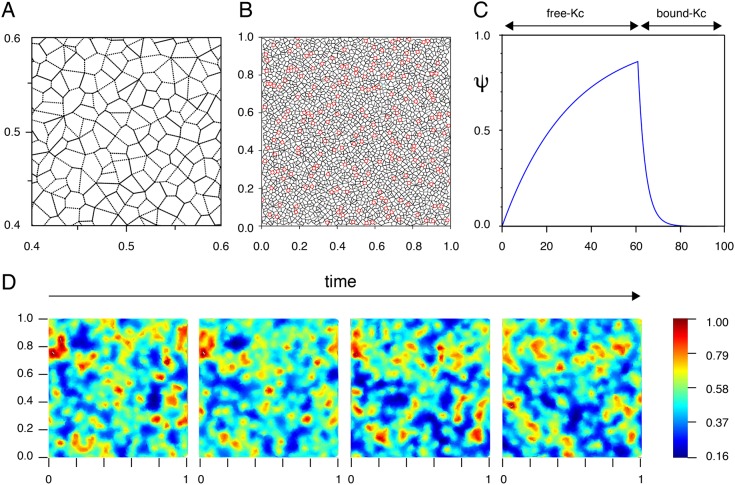
**Melanoblasts are moving on the edges of Kcs that cycle between free and bound states.** (A) Snapshot of a polygon meshwork built as a Voronoi-like diagram for a random set of points, representing the basal keratinocytes that are in contact with the basement membrane. One side of the square corresponds to a length of 130 μm. (B) Complete polygon meshwork and snapshot of melanoblast positions, represented as empty red dots, that are initially randomly positioned on the polygon vertices of the Voronoi-like diagram (in black). One side of the square corresponds to a length of 635 μm. The square is composed of about 4000 Kcs. (C) Time variation of Kc attractive potentials, Ψ, of free Kc and bound Kc. A bound Kc binds a Mb, whereas a free Kc does not. Ψ increases to reach a threshold until a contact with a Mb is made then decreases thereafter. (D) Color representation of Φ_*A*_ potential evolution with respect to time during four simulation steps (for a total of 20 steps). A gradient color scale of the potentials is shown on the right. Red represents a high potential of attraction and blue a low potential of attraction. One side of the square corresponds to a length of 635 μm. For simplification, Kcs are not represented.

The simplest way is to represent Mbs as dots moving on a regular grid of Kcs (Mort et al., 2016). In order to keep both the geometric properties and the natural randomness of cellular shapes in the epidermis, we represent Kcs as polygons that partition the square (Fig. 2A and Appendix S1). The polygons are first built as the Voronoi-like polygons of a set of points (Bock et al., 2010). Mbs are modeled as points (represented as dots) whose positions are the polygon vertices and which are moving along the polygon edges (Fig. 2B). We do not represent the shapes of Mbs and their protrusions, which are extremely variable during their displacements between Kcs. But we can further attribute geometric properties to the Mbs to use them in the interactions with Kcs and Mbs by taking into account the protrusions.

To summarize, Mbs are represented as dots moving on the edges of a diagram defined by a partition of a square-shaped domain into polygons modeling the Kcs. Considering the graph defined by the polygonal tiling (Fig. 2B), at each time step the Mbs occupy a node and have to move (or not) along an edge to an adjacent node. The displacements are a consequence of the interactions of Mbs with Kcs, and Mbs with Mbs, as detailed below.

#### Dynamic interactions between Mbs and Kcs

The migration of melanoblasts between keratinocytes during mouse embryonic development is not fully understood. This migration depends on the surroundings, limited to Kcs (cell non-autonomous), on the coordination between Kcs and Mbs for the movement of Mbs, and on the Mbs themselves (cell-autonomous). According to our current knowledge, the following features have to be included to model the migration of Mb in mouse epidermis.

##### Status of free Kcs and Mb-bound Kcs

Kcs are either directly interacting (bound Kcs) or not (free Kcs) with Mbs. We hypothesized that free Kcs produce soluble diffusible factors that generate a gradient of attraction for Mbs. For example, the Steel, Edn1/3 or/and other ligands produced by free Kcs interacting with Kit, EdnrB or/and other receptors present at the surface of Mbs could be one of these factors. Steel is produced by Kcs and exists in two forms: soluble and membrane bound. One hypothesis is that once Kit interacts with a membrane-bound Steel, a signal is transmitted to the Kcs to stop producing soluble ligands that attract Mbs. Another might be that an Mb could deplete the soluble ligands locally and thus cause an area of relative repulsion. In support of this idea of repulsion, we never observed a Kc interacting with two Mbs on histological skin sections of embryos at various stages.

In the model, we attribute to each Kc a ‘potential of attraction’, Ψ, that can be mainly interpreted as a density of these soluble factors. The production of such diffusible factors would stop once an Mb is in direct contact with a Kc (switching from a free Kc to a bound Kc). In consequence, the extent of attraction of a Kc for a Mb increases with time until a bound Kc is generated (Fig. 2C).

For a free Kc, we assume that Ψ is increasing with respect to time but is bound by a threshold Ψ_*max*_, with a time evolution described in Fig. 2C,D. Thus, the potential falls between 0 and Ψ_*max*_. For a bound Kc, the potential is decreasing with respect to time (Fig. 2C,D and Appendix S1). The speeds with which the potential of attraction of a free (f) or bound (b) Kc for Mb increase or decrease are defined by the coefficients *C*_*f*_ and *C*_*b*_, respectively.

Each keratinocyte generates its own potential of attraction (Ψ) and each Mb senses the result of all potentials of attraction. A potential of attraction, Φ_*n*_(*t*), is calculated at each node ‘*n*’ as an average of the potentials of Kc around the node, and the potential across a field therefore evolves with time (Fig. 2D).

##### Attraction of Kcs for Mbs according to their cell cycle

The production of attracting ligands is certainly dependent on the cell-cycle status of the Kcs. We formulated this hypothesis as Steel production is not constant (Jin et al., 2002). Knowing that all Ks are cycling during embryonic development, the size of the Kcs is linked to the cell-cycle phase (Fig. 2A). Therefore, the size of the Kcs is correlated with the intrinsic potential of attraction for Mbs. In the model, a simple way to take into account the size of a Kc in the attractiveness of a Mb is to modulate, for each Kc, the potential threshold Ψ_*max*_ by adding a number proportional to the size of the associated polygon; this value remains constant during the time of analysis (equivalent to three hours). We chose to measure the size by the perimeter of the polygon, which is easy to calculate.

##### Migration of Mbs between Kcs

**Fig. 3.**
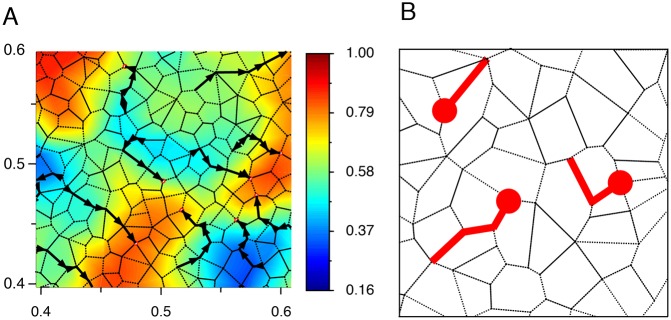
**Melanoblasts feel the potential of attraction of Kcs (Φ_*A*_) with their protrusions and move on the edges of Kcs.** (A) Color representation of Φ_*A*_ Kc potentials superimposed on a Voronoi-like diagram. The isovalues of the opposite of the potential at the last step are displayed with colors. Gradient color scale of the potentials is shown on the right. Mbs are represented as empty red dots moving on the edges of the polygons modeling the Kcs. Five steps of Mb movement are shown (thick black arrows). (B) Graphical representation of one, two and three Mb protrusions in red, along the edges of Kcs.

Melanoblasts migrate along the basement membrane between basal keratinocytes. Basal Kcs interact with the basement membrane and are strongly cohesive with surrounding Kcs. In order for Mbs to migrate through Kcs, cell-cell adhesions (including E-cadherin and its associated temporal dynamics) of Kcs have to be disrupted and cell-matrix adhesion (including integrins) have to be modified to create the path between two Kcs (Fig. 3A). In this model, we do not include this unknown parameter but it is directly linked to the average speed of each Mb. The mathematical model imposes that each Mb has to be located on a node at each time step. In consequence, the average speeds of the Mbs are all the same and are equal to the polygonal edge mean length divided by the time step. But *in vivo* Mbs have various velocities. To include this property at each time step, we add that each Mb has the possibility of moving or not. A probability (P), depending on the Mb, is then assigned to each Mb for its movement at each time step, with *P*_*min*_≤*P*≤1. *P*_*min*_ controls the standard deviation of the Mb speed (see Appendix S1).

##### Cell-cell sensing of melanoblasts by other melanoblasts and keratinocytes

Melanoblasts are composed of a cell body and several protrusions of different sizes and diameters. The cell body and the major protrusions are easily observable under a confocal microscope, but the protrusions possessing a low amount of fluorescence are not, owing to a lack of sensitivity. In this respect, it is difficult to evaluate the full area covered by a Mb. To simplify this aspect, we hypothesized that the maximum number of primary and secondary protrusions from a Mb is approximately five and the maximum size of a protrusion corresponds to two Kcs (Fig. 3B). For simplification of the model, we considered that there is only one protrusion per melanoblast with different sizes.

In the model, the protrusions and their lengths are modeled in the Mb-Kc interactions by taking into account the attracting potential not only at all adjacent nodes but also at the adjacents of the adjacent nodes up to two Kcs away. At each adjacent node of a specific Mb position, a resultant attractive potential Φ_*A*_ is calculated with the information arising from all Kc potentials at the nodes attained by the protrusions. An isolated Mb should first head toward the adjacent node with its maximum potential, but a Mb can be repelled by the presence of a surrounding Mb (see Appendix S1).

##### Distribution of Mb among Kc and their velocities

Melanoblasts are uniformly dispersed among basal keratinocytes located at the basal layer of the epidermis. The uniform distribution of Mb is warranted at the first step by a random choice of their position and we hypothesized that in the other steps it could be due to a physical repulsion between two Mbs and to some regularity property of the attraction potential of Kcs. The repulsion can be modeled by a new repulsing potential Φ_*R*_ added to the Kc attractive potential Φ_*A*_ (see Appendix S1). The action of one Mb cannot be isolated. In fact, if one Mb is in contact with a Kc, the potential of this Kc falls (Fig. 2C) regardless of the positions of other Mbs; however, the attracting potential decreases more slowly.

We also assume that a Mb cannot occupy, during some time delay, a position previously occupied by another Mb. To add this assumption into our model, the potential Φ_*R*_ of repulsion between Mbs is modified by taking into account not only the last position of a Mb (in this case the potential is equal to 0 or 1) but also the earlier positions of the Mb with a decreasing factor: Φ_*R*_=*ρ*^*k*^ after *k* time steps (*ρ*<1). After some comparisons between computed and experimental pictures of trajectories, we determined that 60 min was the average time that the presence of a previous Mb could impede the arrival of a novel Mb at the same position, which can be defined as a ‘repulsion trail’.

Finally, according to the analysis of the experimental movies (Movies S1-S9), there is sometimes a clear anisotropy of the Mb trajectories: one direction can be favored. To take this property into account, a potential Φ_*D*_ can be added in order to favor one direction. This potential depends on the angle between the displacement direction and the anisotropy direction with a weight *w*_*D*_ to calibrate (see Appendix S1).

#### Complete Kc potential

To sum up all of the previous assumptions, a total potential Φ_*n*_(*t*) at the node number *n* at time *t* is calculated by summing the Kc potential Φ_*A*_, the repulsive Mb potential Φ_*R*_ and the anisotropy potential Φ_*D*_ with weights *w*_*R*_, *w*_*D*_ to determine
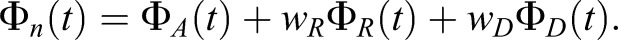
Here, Φ_*A*_ is the attracting Kc potential calculated by an accumulation of potentials of neighboring free or bound Kc, which themselves depend on the time. Φ_*R*_ is the Mb repulsive potential, varying between 0 and 1, according to whether the adjacent nodes are free or occupied, and Φ_*D*_, varying between 0 and 1, is introduced, if needed, to favor one direction.

Finally, whether a Mb heads, at time *t*, from a specific node towards the adjacent node numbered *n*, which has the maximum potential Φ_*n*_(*t*) or remains at the current specific node, depends on a given probability specific to each Mb (a ‘deterministic model’ with variable Mb mean speeds). To this probability, we add the possibility to choose to migrate randomly, with a fixed probability *P*, toward any adjacent node (a ‘partially’ or ‘totally’ random model according to *P*>0 or *P*=1).

In summary, four steps are followed to allow the simulation of the movement of the Mbs. First, Kc paving is established. Second, Mbs are randomly placed on Kc polygons vertices. Third, 10 unrecorded stationary steps are simulated to establish the initial map of attractive and repulsive potentials, so that we model a dynamic system already at equilibrium. This corresponds to a dynamic process in a ‘stationary’ state, which is a dynamic process where the initial state has no more influence on trajectory mean properties. Fourth, a further 20 steps are simulated such that cells move a similar mean distance to their experimental counterparts.

#### Geometric characteristics of Mb trajectories

In this section, we consider trajectories without taking into account their geometrical start point. The goal is to first generate the simulated trajectories prior to comparing them with the experimental trajectories. The various parameters of the algorithm are modified in order to maximize the match between experimental and simulated trajectories. Each simulation uses a few parameters that are assessed to simulate a given experiment (*w*_*D*_ and *P*_*min*_) and other parameters, which have been fixed in order to reflect the general properties of Mb trajectories (Table S1). Of course, experimental and simulated trajectories cannot match exactly due to many random factors: the initial Mb positions, the exact geometry of Kc tissue and the biological intrinsic randomness. Therefore, we can only compare qualitative or average properties of the trajectories.

To start, we use classical criteria to evaluate experimental or simulated trajectories: the distance of migration; the Euclidian distance, defined as the length between the point corresponding to the start and end of the migration; the directionality of cell migration, defined as the ratio of the Euclidian start-end distance over the total distance; and the average speed of migration, corresponding to the total distance of migration divided by the time associated with this migration.

**Fig. 4.**
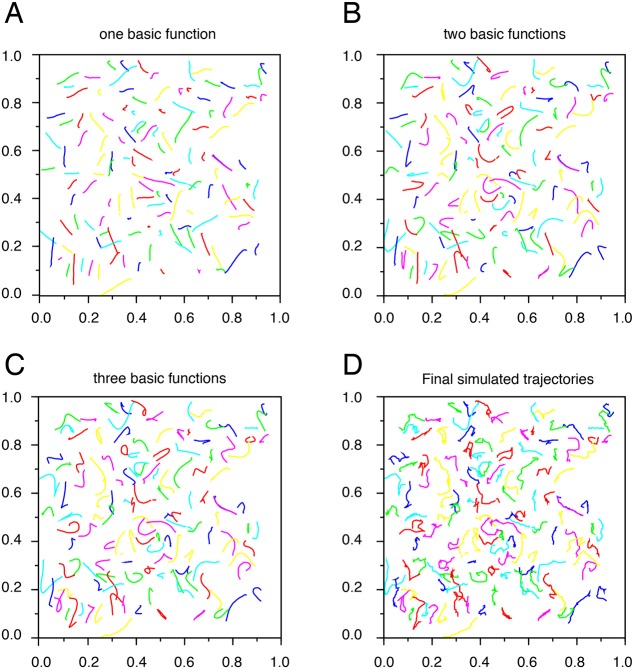
**Each trajectory is written as a complex linear combination of at least three basic trajectories.** (A-C) Graphical representation of simulated Mb trajectories based on one (A), two (B) and three (C) basic functions. (D) Graphical representation of final Mb simulated trajectories that take into account all properties of the trajectories being a complex linear combination of the basic trajectories.

Then, we aimed to quantify various qualitative geometric properties of the curves, such as main direction, smoothness, return points and circularity (Fig. 4 and see Appendix S1). After having defined a few basic trajectories in an optimal way, each trajectory is written as a complex linear combination of these basic trajectories. Since multiplication by a complex number is a similitude (i.e. a rotation and a dilatation), each trajectory is a linear combination of trajectories similar to the basic ones. The coefficients of these basic trajectories give information on the shape and smoothness of the trajectories. By rebuilding the trajectories with only one basic function, one gets the main direction of the trajectories (Fig. 4A). Two basic functions also show curvatures: accelerations and return points (Fig. 4B). With three basic functions, the trajectories can also exhibit circularity (Fig. 4C). Moreover, the decreasing speed of the coefficients shows the general smoothness of the trajectories: slowly decreasing coefficients characterize random trajectories whereas quickly decreasing coefficients define simple curves. This analysis is similar to Fourier series decomposition (Iwata and Ukai, 2002) but with basic functions, which are best adapted to fit a given set of trajectories. Once all functions are taken into account, final simulated trajectories are generated (Fig. 4D).

**Fig. 5.**
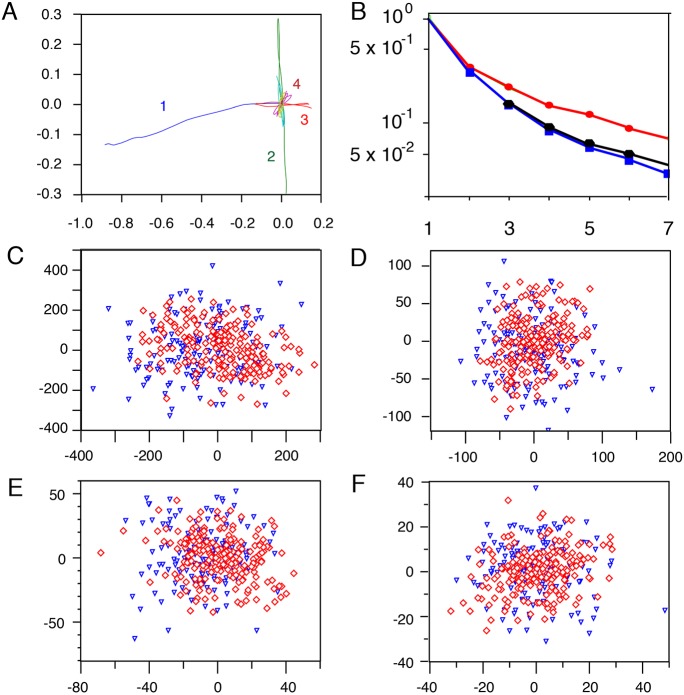
**Comparison of experimental and simulated sets of trajectories with the help of their components in a common basis.** (A) Graphical representation of the four main basic functions extracted from experimental trajectories. (B) Comparison of Mb trajectories with the help of their components in a common basis: experimental (blue squares), simulated (black hexagons), random with Mb repulsion (red circles). *x*-axis, index of the components; *y*-axis, mean value of basis coefficients modulus in logarithmic scale. For each comparison, the trajectory basis is determined by the experimental set of trajectories. (C-F) Graphical representation of complex coefficients of the four first basic trajectories for the experimental (blue triangles) and simulated (red lozenges) trajectories. The distance from the origin of coordinates to a marker is the weight of one basic trajectory in the representation of the corresponding trajectory. The almost isotropic distribution of markers shows the almost isotropy of trajectories. C is the first basic trajectory, D the second, E the third and F the fourth basic trajectory. The scales are decreasing from the first basic trajectory from −400 to +400 to the fourth basic trajectory from −40 to +40.

The above method was used to extract basic functions from experimental trajectories (Fig. 5A). Both sets of trajectories (experimental and simulated) are expanded into this basis and then can be easily compared (Fig. 5B). Using the fact that the four first coefficients bring a thorough analysis of the shape of a set of trajectories, we can compare the simulated and experimental trajectories (Fig. 5C-F).

### Challenging the mathematical model

From a series of photomicrographs, a set of Mb displacement trajectories was extracted by manual tracking. Our aim was to check biological assumptions and to assess the parameters of the simulation in order to make both sets of trajectories (experimental and simulated) similar. In the sections below, we compared the experiments with simulations performed with different hypotheses. All simulations were performed with the following biological assumptions: two Mbs cannot occupy the same node at the same time, and the Mb velocity distribution is obtained with the method described above.

We compared the following features between the experimental and simulated trajectories: (1) the total distance of migration; (2) the average speed of migration; (3) the Euclidian start-end distance; (4) the directionality of cell migration, which is defined as the ratio of the Euclidian start-end distance over the total distance; and (5) the coefficients of the expansion in the basis defined by the experimental trajectories as described above.

#### Analysis of biological trajectories

**Fig. 6.**
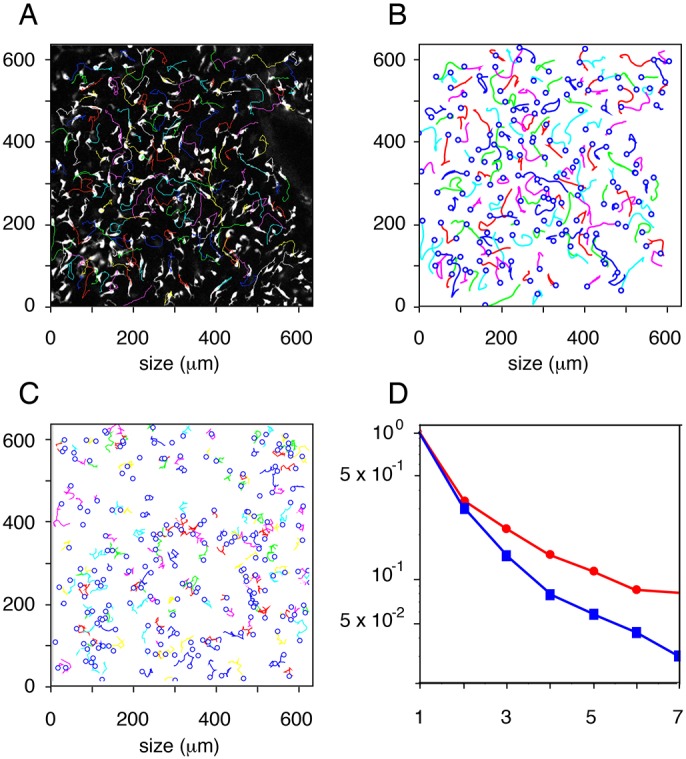
**Comparison of biological trajectories with simulated trajectories generated with a random displacement model.** (A) Superimposition of the trajectories of each tracked cell in Movie S2, with a snapshot of the last picture of the movie. (B) Graphical representation of Mb trajectories in Movie S2. Empty blue dots represent the start position of each tracked Mb. (C) Graphical representation of Mb trajectories generated by the mathematical model with the random displacement model. Empty blue dots represent the start position of each Mb. (D) Comparison of Mb trajectories with the help of their components in a common basis: experimental (blue squares) and simulated with random (red circles). Note the profound difference between the two curves. *x* and *y* axes represent the size in μm of the acquisition window.

Time-lapse imaging of live cells from Tyr::CreA/°; ZEG/° embryo skin explants driving melanoblast-specific expression of GFP was performed to follow the movement and the trajectories of fluorescent Mb. An example of the experimental trajectories of Mb is shown (Fig. 6A,B). The doubling time of melanoblasts *in vivo* was estimated to be 18 h (Luciani et al., 2011). The trajectories of the melanoblasts were followed for 3 h. During this period, the number of mitoses is limited and the main physiological constraints are preserved and sufficient to evaluate the complexities of the movement, including pluri-directional movements, acceleration and/or pauses.

#### Model with random displacements

We use a simple random displacement model based on a biologically natural assumption: at each time step a Mb located at a node can jump from this node to one of the randomly chosen free adjacent nodes of the Kc polygonal network (in the model described above it is named ‘totally random’). An example of the simulated random trajectories of Mbs is shown (Fig. 6C) in addition to the comparison of expansion coefficients of simulated and experimental trajectories (Fig. 6D). The trajectories generated with random displacements are totally different from the experimental trajectories. In conclusion, the Mb does not migrate randomly.

#### Model driven by Kc and Mb potentials

**Fig. 7.**
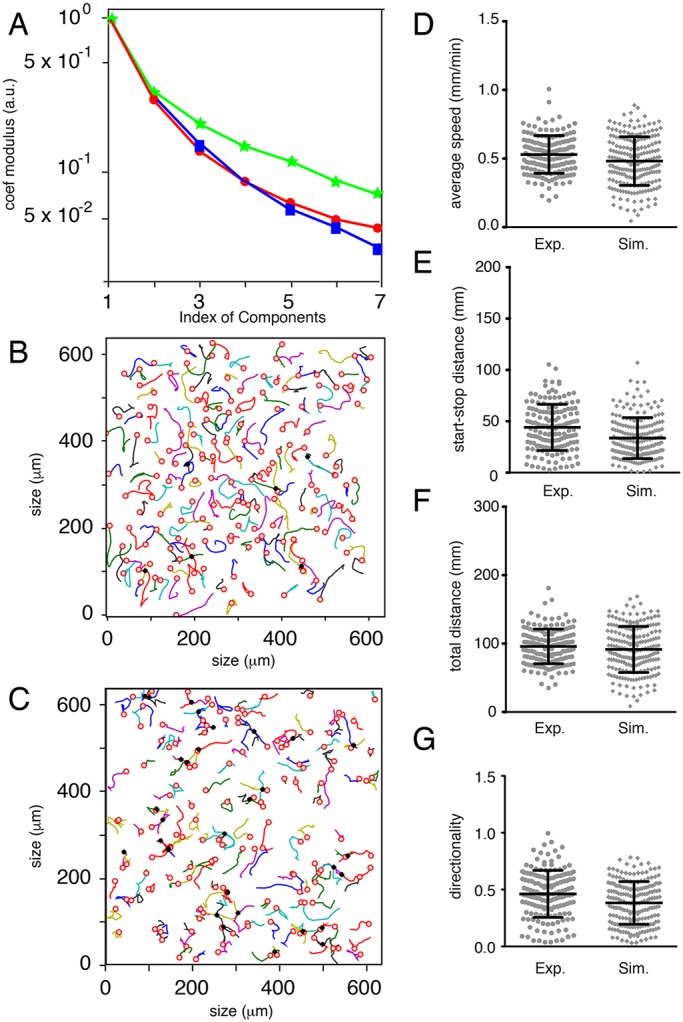
**Comparison of biological trajectories with simulated trajectories generated with a random deterministic model.** (A) Comparison of Mb trajectories by their components in a common basis: experimental WT2 (blue squares and line), simulated with random (green stars and line), simulated with the help of Kc-Mb attraction and Mb-Mb repulsion potential (red circles and line). *x*-axis, index of the components; *y*-axis, mean value of the basis coefficients modulus in logarithmic scale. The blue and red curves are very similar. (B) Graphical representation of 155 Mb trajectories from Movie S2 presented in Fig. 6A with crosses to exhibit the six trajectory crossings. (C) Graphical representation of 157 simulated trajectories with crosses to exhibit the 31 crossings. (D) Average speed of Mbs extracted from Movie S2 (Exp.) and the mathematical model (Sim.) from the retrieved sets of coordinates. (E) Euclidian distance of Mb trajectories extracted from Movie S2 (Exp.) and the mathematical model (Sim.) from the retrieved sets of coordinates. *N*>170 Mb for Exp. and Sim. (F) Total distance of Mb trajectories extracted from Movie S2 (Exp.) and the mathematical model (Sim.) from the retrieved sets of coordinates. *N*>170 Mb for Exp. and Sim. (G) Directionality of Mb trajectories extracted from Movie S2 (Exp.) and the mathematical model (Sim.) from the retrieved sets of coordinates. *N*>170 Mb for Exp. and Sim. Statistics were performed using the non-parametric Mann–Whitney *t*-test: (D) *P*=0.0127, (E) *P*<0.0001, (F) *P*=0.2575 and (G) *P*=0.0005.

We next employed the ‘deterministic model’, where we integrated the potential of attraction of Kcs (Φ_*A*_) and the simple repulsion between Mbs (Φ_*R*_), taking into account only the neighboring nodes for repulsion. The parameters (number of Kcs, number of Mbs, average size of Kcs) used for the simulations are identical for the experimental and simulation movements (Table S1). The expansion coefficients of simulation and experimental trajectories are very similar (Fig. 7A), and the characteristics of the trajectories (speed, Euclidian distance, total distance and directionality) are also similar (Fig. 7D-G and Fig. S1A-D). In this analysis, we performed a translation of all trajectories from the same origin, thus disregarding their geometrical start points. Surprisingly, the visual observation of the trajectories reveals a major difference: the biological displacements appear to be dependent on each other (Fig. 7B), as they are locally more parallel to each other than in the case of the Mb-simulated movements (Fig. 7C). This property/observation is difficult to quantify but is linked to the number of intersections between trajectories; for example, during the same period of time estimated to 1 h, one may observe six crossings for a specific biological experiment and 31 for the simulation (Fig. 7B,C; see Appendix S1). Similar analyses were performed with independent biological experiments, revealing the exact same phenomenon (data not shown). In conclusion, according to a series of criteria, with our first assumptions the simulation fits very well with the biological evidence, but it is not fully satisfactory.

#### Model driven by Kc and Mb potentials with memory

**Fig. 8.**
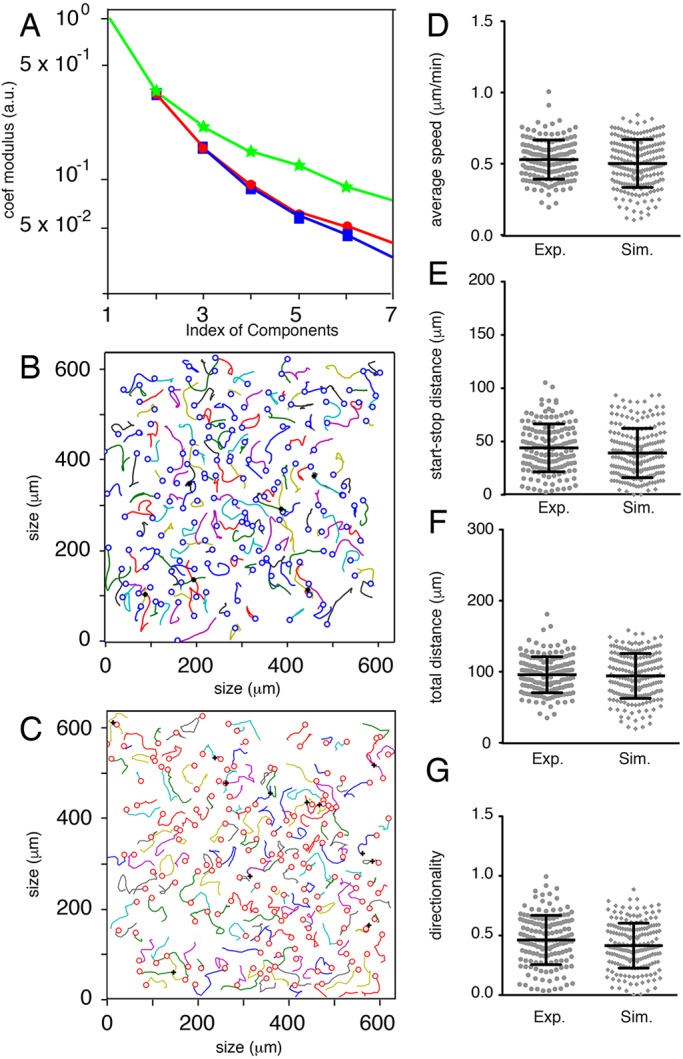
**Comparison of biological trajectories with simulated trajectories generated with a random deterministic model, including a memory parameter.** (A) Comparison of Mb trajectory properties with the help of their components in a common basis (see ‘Geometric characteristics of Mb trajectories’): experimental WT2 (blue squares and line), simulated with the same hypothesis as that in Fig. 7 and adding the non-overlapping hypothesis (red circles and line) or random with Mb repulsion (green stars and line). *x*-axis, index of the components; *y*-axis, mean value of the basis coefficients modulus in logarithmic scale. The experimental sets are described (see ‘Analysis of biological trajectories’). For each comparison, the trajectory basis is determined by the experimental set of trajectories (see ‘Geometric characteristics of Mb trajectories’). (B) Graphical representation of 155 Mb trajectories from Movie S2. Black crosses indicate the crossings between two trajectories. (C) Graphical representation of 170 simulated trajectories with 12 crossings. (D) Average speed of Mb extracted from Movie S2 (Exp.) and the mathematical model (Sim.) from the retrieved sets of coordinates. (E) Euclidian distance of Mb trajectories extracted from Movie S2 (Exp.) and the mathematical model (Sim.) from the retrieved sets of coordinates. (F) Total distance of Mb trajectories extracted from Movie S2 (Exp.) and the mathematical model (Sim.) from the retrieved sets of coordinates. (G) Directionality of Mb trajectories extracted from Movie S2 (Exp.) and the mathematical model (Sim.) from the retrieved sets of coordinates. Statistics were performed using the non-parametric Mann–Whitney *t*-test: (D) *P*=0.2157, (E) *P*<0.0281, (F) *P*=0.8649, (G) *P*=0.0238.

We included a memory parameter that prevents a Mb from crossing the trajectory of another Mb, defined as repulsion trail. A novel repulsion potential Φ_*R*_=*ρ*^*k*^, *ρ*<1 has been included at each node previously occupied by a Mb, which has a decreasing effect with time step *k*. As expected, the expansion coefficients of simulation and experimental trajectories are again very similar (Fig. 8A), and the characteristics of the trajectories are optimal (Fig. 8D-G, Figs S2-S5). Moreover, the number of crossings is similar in both cases (Fig. 8B,C). Comparisons between other experiments and simulations were performed with the same model (Figs S3-S5). Hence, the inclusion of the repulsion potential (*ρ*^*k*^) leads to similar biological and simulated trajectories.

**Fig. 9.**
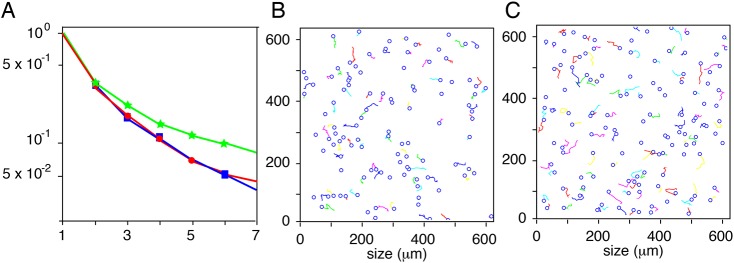
**Comparison of Rac1 melanoblasts trajectories with simulated trajectories.** (A) Comparison of Rac1 Mb trajectories with the help of their components in a common basis: experimental (blue squares and line), simulated with random (green stars and line), simulated with the same model as in Fig. 8 but with repulsion potential taking into account only adjacent nodes to model short protrusions (red circles and line). *x*-axis, index of the components; *y*-axis, mean value of basis coefficients modulus in logarithmic scale. The blue and red curves are very similar. (B) Graphical representation of 106 Rac1 Mb trajectories. (C) Graphical representation of 127 simulated Mb trajectories.

Finally, we wished to test the generality of our model. In order to show that it was robust to serious changes in other aspects of cell migration, we tested its ability to accurately represent the trajectories of melanoblasts lacking Rac1 (Tyr::Cre/°; Rac1 f/f). These cells have been shown to be slower and fewer in number, and have shorter protrusions (Li et al., 2011). We account for the first two changes by similar methods to the wild-type data. In order to model the third, we only allow cells to sense adjacent sites when calculating Φ_*n*_. We decomposed the trajectories of experimental Rac1 cells into basic functions. Making only the stated changes to the model, we were able to accurately recapitulate the observed difference in Mb trajectories, demonstrating that the model maintains predictive power even after serious perturbation of the experimental system (Fig. 9A-C).

## DISCUSSION

The population of the embryonic skin by melanoblasts is a fundamental example of developmental individual cell migration and has been an active subject of study for many years. The mechanisms driving melanoblast migration from the neural tube and through the dermis and epidermis are complex, and the molecular mechanisms are increasingly revealed using new methods such as RNA-seq and live-explant imaging (Colombo et al., 2012; Koludrovic et al., 2015; Mort et al., 2014; Woodham et al., 2017). However, to fit together the huge amount of information that we have about this biological system, we need models to predict properties of the movement so that we can predict the effects of perturbations and gain a deep understanding of developmental cell migration in general.

Here, we present a model for how Mbs distribute themselves in the epidermis of the mouse skin, essentially the penultimate step in their migration from the neural crest before they reside in hair follicles in the adult. Biological observations provide information on the domain size, the mean Kc size (or equivalently the number of Kcs), the average length of Mb trajectories (and the statistical dispersion of these lengths) and the total moving time (or equivalently the average Mb speed). These parameters were used to calibrate some simulation parameters: the domain size, the mean size of polygonal edges, the real duration of a numerical time step (recall that the numerical time step corresponds to a jump along a polygonal edge) and a random parameter to model the natural dispersion of Mb velocities (in the simulation a Mb moves or does not move according to a random number). However, parameters of the variations of Kc potentials and the duration of the repulsion trail memory effect were not directly known. Our model reveals several features that had been previously predicted, such as attraction of melanoblasts to keratinocytes and repulsion/exclusion of melanoblasts from each other. However, our model also reveals an unexpected feature: melanoblasts remember the pathways that they traverse in the skin and do not cross over previously traversed pathways. This leads to an exciting feature whereby they behave more collectively than was thought and suggests that additional signals – in the matrix, on the keratinocytes or in the soluble spaces – direct migration and allow memory. This feature could be mediated by Kc producing membrane-bound or soluble Steel, and Mbs either consuming these or regulating the expression levels in the Kcs by direct contact. Alternatively, loosening of the cell-cell contacts between Kcs could be caused by Mb migration and this may be inhibitory to further Mb migration for a period of time until these cell-cell contacts recover.

The good match between the simulated and the biological results have only been obtained after calibrating the abstract parameters in a natural way, as explained above, and after a careful choice between several mathematical hypotheses modeling the biological assumption (see Appendix S1). Recall that the average speed of migration and total distance traveled within the simulations match their experimental counterparts by definition: for the mathematical model, the first number determines the time step duration in a simulation and thus the average total distances are equal because the time intervals for the studies are the same. A good match (evaluated according to the *P*-value: the higher it is, the better the match is) of the s.d. of average speeds is obtained by a suitable choice of the moving probability assigned to each Mb. The expansion coefficients show the complexity of any trajectory. The greater the coefficient modulus is, the more oscillatory is the trajectory. Hence, the decreasing speed of the coefficient modulus in logarithmic scale is a good estimate of the similarity of the trajectories. However, with the same coefficient modulus but different complex arguments, two trajectories can have different shapes: e.g. either an oscillatory trajectory or a trajectory with return points.

In conclusion, the general deterministic mathematical model simulating the Mb displacements presented in ‘Complete Kc potential’ used with a suitable choice of biological assumptions, mathematical hypotheses and parameters, enables the stimulation of Mb displacements in a Kc tissue without extra random hypotheses except for the geometric description of Kcs. The main biological assumption is that a Mb is attracted by a factor, or factors, produced by the Kc that diffuse in the tissue and that a Mb bound to a keratinocyte induces a reduction of production of such factors. The repulsion/exclusion between Mbs ensures a regular distribution of Mbs. An additional hypothesis had to be introduced in order to retrieve certain correlations between trajectories associated with the number of crossings between trajectories: the tracks of a Mb repel other Mbs for a while. The discovery of this repulsion suggests an exciting way for biological systems to use chemical or physical memory to drive coordinated behavior of groups of single cells. Uncovering the basis for the repulsion trail will no doubt reveal interesting mechanisms controlling developmental migration.

## MATERIALS AND METHODS

### Transgenic mice

All experiments were performed according to UK Home Office regulations. To investigate the migration of melanoblasts *in vivo*, mice carrying a Z/EG double reporter transgene (Novak et al., 2000) were crossed with tyrosinase CreA [Tyr::Cre, JAX Stock 029788 B6.Cg-Tg(Tyr-cre)1Lru/J (www.jax.org/strain/029788)] mice on a C57BL6/J background (Delmas et al., 2003) to drive GFP expression in the melanoblast lineage. To investigate the functions of Rac1 in melanoblast migration *in vivo*, mice carrying a Z/EG double reporter transgene were crossed onto the Tyr::Cre/°; Rac1 f/f background to drive GFP expression in the melanoblast lineage. Movies S1-S6, S9 from wild-type and Tyr::Cre/°; Rac1 f/f embryos were analyzed. Some of the movies have been used for analysis previously (Li et al., 2011), but the analysis here was carried out completely independently of previous analysis.

### *Ex vivo* imaging of melanoblast migration

The experimental set up was as adapted from Mort et al. (2010). E15.5 embryos were harvested and dissected, removing a section of the embryonic skin containing the dermis and epidermis. The sample was sandwiched between a nuclepore membrane (Whatman) and a gas-permeable Lumox membrane in a 24-well Greiner Lumox culture dish (Greiner Bio-One) so that the epidermal side of skin was in contact with Lumox membrane. Matrigel (BD Bioscience) was used to cover the membrane and the plate was incubated at 37°C for 10 min. Culture medium [Phenol Red-free DMEM supplied with 10% FBS and 100 mg/ml primocin (InvivoGen)] was added. Time-lapse images were captured using an Olympus FV1000 or Nikon A1 confocal microscope in a 37°C chamber with 5% CO_2_ for 3 h. Individual fluorescent Mbs were tracked manually using the Manual Tracking plug-in for ImageJ developed by F. Cordelières (rsbweb.nih.gov/ij/plugins/track/track.html), with distance/time measurements taken every 5 min for 3 h. The migration speed, the total distance of migration, the Euclidian distance of migration and the directionality of migration were extracted from the coordinates retrieved from the manual tracking plug-in. Mean values±s.d. and statistical analyses were calculated and plotted using Graphpad Prism (Graphpad Software) and significance was determined using two-tailed Mann–Whitney *t*-tests (after determining the non-Gaussian distribution of the data using the Shapiro-Wilk normality test).

## Supplementary Material

Supplementary information
