# Supplementary material for: Simulation of melanoblast displacements reveals new features of developmental migration
Source: Development. 2018 Jun 11;145(12):dev160200. doi: 10.1242/dev.160200 (PMC6031402; doi:10.1242/dev.160200)
Supplement: Supplementary information [file develop-145-160200-s1.pdf]

Appendix S1. Model description

## Building the geometric model

First we built the positions of the "centers" of the Kc. Because the Kc dimensions are only slightly different, making a random choice of the coordinates does not lead to an adequate center distribution, so we used the Halton method to generate points with regular density {Halton, 1960 #3099}. We added a slight modification using a small random displacement of the points to avoid some point alignments. Then we built the Voronoi diagram based on these points, but the result was not satisfactory because some edges were very small. For this reason, we deleted all edges smaller than a given length $d_{min}$ and we merged the corresponding vertices. Let us notice that a genuine Voronoi diagram has, in our context, the spurious property that almost all vertices belong to only three edges, which was no longer the case after removing the small edges. For our simulations, the maximum of random displacements is equal to 0.2 µm and the minimal edge length is 0.3 µm, both with respect to the mean distance between centers. In order to avoid dealing with boundary conditions, the domain of study is in fact a periodic square, meaning that a melanoblast moving out of one square side enters the opposite side. Hence, the Voronoi diagram is also periodic: the nodes of the upper side of the square domain are connected to the nodes of the underside and those of the left side to the nodes of the right side.

## Potential functions

We assign to each Kc a "potential" $\Psi(t)$ at time $t$, which can be interpreted as a factor density but which can also have an abstract mathematical meaning of the capacity of attraction of Mb by Kc. For a free-Kc, we assume that the time evolution of $\Psi$ is modeled by the differential equation $\frac{d\Psi}{dt} = C_f(\Psi_{max} - \Psi)$. For a bound-Kc, the time evolution of the potential is modeled by the differential equation $\frac{d\Psi}{dt} = -C_b\Psi$.

These Kc potentials enable us to define a density of factors by a diffusion process of the factor. At each time step the Kc potential diffusion is obtained by repeating (up to three times) the following process:

- At each polygon vertex, a potential is calculated by an average of the potential of the Kc to which the vertices belong.
- The new Kc potential is the average of the potential of all its vertices.

Thus the factor is propagated over three Kc away after which a value for the potential is computed as a weighted average of the initial value and of the new one. If the weight $v_d$ is 0, there is no diffusion and if it is 1, the diffusion is maximal. This density of factors enables to calculate a potential of attraction $\Phi_A(\mathrm{t})$ at each node $n$ as an average of potentials of Kc lying around the node $n$. The potential of repulsion between Mb, denoted $\Phi_R(t)$, computed at a given node $n$, is equal to 0 or 1 according to the fact that all nodes adjacent to $n$ are occupied by another Mb or not. To model the additional assumption that a Mb can repulse another Mb for a short time after it has left a node, we consider more generally that at a given time, a node occupied by a Mb $k$ time steps before this tim$e$ has a potential $\rho^k$ where $0 < \rho < 1$. Therefore, the potential $\Phi_R(t)$ is computed at a node $n$ as the minimum of the potential of all nodes adjacent to $n$ (with the possibility of considering several layers of adjacent nodes).

Development • Supplementary information

Some of the experimental sets of trajectories we want to simulate exhibit a clear anisotropy of the trajectory directions without a specific orientation along this direction, possibly due to some experimental and non-biological cause. To take this anisotropy into account, a term $\Phi_D(t)$ is added to the potential in order to favour one direction. This term is proportional to the absolute value of $\cos\theta$, where $\theta$ is the angle between the displacement direction and the favoured direction.

## Melanoblast velocity

The mean speed of a Mb is the trajectory length divided by the time duration, and in a simulation only the number of time steps is known but not the duration of a time step. Thus, we determine the time step duration in order that the average of all Mb mean speeds is equal to a given experimental value. Let us remark that if the number $N$ of time steps is large enough, all of the trajectory lengths will be almost equal to $NL$, where $L$ is the mean value of the Kc polygon side lengths. In this case, the distribution of lengths is very narrow and only depends on geometric properties (e.g. the Kc polygonal shapes). However, the trajectory lengths must also depend on biological features of the Mb. The classical way to correct this model drawback is to allow for the Mb sometimes to be stationary. That is why in order to obtain a realistic distribution of Mb mean speeds the following process is added: a Mb numbered $m$ is assigned a probability of moving $p_m$ with a set of probabilities $p_m$ uniformly distributed between a value $p_{min}$ and 1. At each time step and for each Mb a uniformly distributed random number $p$ is chosen between 0 and 1 and the Mb is moving if $p > p_m$. Hence the number $p_{min}$ controls the standard deviation of Mb mean velocities. The value $p_{min}$ has been calibrated about 0.3 but it can depend on the simulation when the aim is to simulate an experiment.

## Trajectory analysis tools

In this section we analyze a set of trajectories by writing them as complex linear combinations of a few basic curves. That is mathematically equivalent to performing a Principal Component Analysis (PCA) on the set of Mb displacement steps, but here it is more intuitive. Keeping in mind that a plane trajectory defined by *N* points defines a complex vector of dimension *N*, a set of trajectories is in fact a set of complex vectors in dimension *N*. The basic trajectories are built using a complex "Singular Value Decomposition" of this set of vectors, a mathematical tool close to a PCA and which can be outlined as follows: the first basic trajectory is the trajectory which, after a similitude (i.e. a rotation and a dilatation), best fits all the trajectories, in the mean square sense. After subtracting this first approximation from all trajectories, we obtain a second set of trajectories. The second basic curve is built in the same way as the first one from this second set of trajectories, and so on. In Figure 4, the application of this analysis for the trajectory of the experiment WT2: the first basic curve is close to a straight line and shows the direction of the trajectory (and its start-end length). The second basic trajectory shows a simple oscillation of the trajectory around this line (that can be also away and return). The third basic trajectory shows a double oscillation and so on (the basic trajectories of Figure 4A have been scaled by a weight proportional to their average contribution to fit the trajectories). This analysis is similar to a Fourier series decomposition but with basic functions which are the best adapted to each set of trajectories. Actually, less than 7 basic trajectories are needed to represent all trajectories with an error less than 2%. To sum up, for each set of trajectories an optimal set of a few basic trajectories is calculated such that each trajectory is a complex linear combination of these basic trajectories. Since a multiplication by a complex number is a similitude (i.e. a rotation and a dilatation), meaning that each trajectory is a linear combination of trajectories similar to the basic ones. Figure 4 shows the rebuilding of a set of experimental trajectories taking account 1, 2 or 3 basic functions.

Development • Supplementary information

Now, to compare two sets of trajectories by the above analysis, basic trajectories are extracted from one set and both sets of trajectories (experimental and simulated) are expanded into this basis. Note that in order to make a meaningful comparison between two sets of trajectories, some preprocessing must be applied to the data. First the time interval must be the same for the two sets; hence we reduce the longest to the smallest. Then, since a trajectory is in fact a set of points, i.e. a complex vector, all the trajectories must have the same number of points. That is why all the trajectories are interpolated with 100 points to prevent the loss of the precision.

**Crossing number assessment**

The aim is to assess the number of crossings of two Mb trajectories, or to be more precise, the number of intersections of the two curves defined by the trajectories. Two irregular curves can have many intersections without a clear meaning of their number of intersections due to the lack of precision of experimental data and calculations. That is why we consider it more meaningful to count the number of pairs of curves that have any intersections. In short we do not count all crossings between two trajectories but only those occurring during the memory effect times and we count at most one crossing between two trajectories in order to avoid that two neighbouring trajectories lead to a lot of crossings. The following method is used: the domain of study (a square) is divided into small squares and we count the number of pairs of curves that are going through a small square, but only once. Notice that this method does not distinguish genuine crossings from one-sided contacts.

**Justification of method choices and parameter assessment**

The geometric parameters (quasi random polygon centers, the ratio of randomness, and the minimal edge length $dmin$) are chosen to mimic the biological data, here mainly the Kc polygonal shape (see Figure 2A). The number of Mb and the density of Kc are the same as in the experiments (about 220 Mb and 4,000 Kc with an average edge size of 10µm).

The main choices are in the Kc potential definition:

- The maximum $\Psi_{max}$ of the Kc potential depends of the Kc polygon size but after some tests we choose to leave it invariable and since its value has no meaningful effect on the trajectories, it is fixed to $\Psi_{max} = 1$.
- In order to get a good match between experiment and simulation, the Kc attracting potential must combine suitable spatial and time variations: both are obtained with a quick potential decreasing for bound Kc and a suitable diffusion for the potential. With other choices the potential is either too flat, leading to spatial concentrations of Mb, or too irregular, leading to too random-like trajectories. The speed of increasing $C_f$ of a free Kc potential, the speed of decreasing $C_b$ of a bound Kc potential and the speed of diffusion of the factors associated with these potentials are strongly correlated with these qualitative properties and after some qualitative and quantitative tests, we calibrated the values $C_f = 0.2$, $C_b = 10$ and $C_f = 2$ and the coefficient weight for the diffusion $v_d = 1$ in order to get a noticeable and spatially consistent potential variation after a few time steps.
- Note that the attractive and repulsive potentials $\Phi_A$ and $\Phi_R$ are of the same order of magnitude, hence the ratio $w_R$ between the Kc potential and the Mb repulsion potential is of the order of magnitude 1. Smaller or larger values lead to neglecting the effect of one of the potentials. The results are not very sensitive to the exact value. The potential $\Phi_D$ favouring a direction has a small coefficient, between 0 and 0.15, depending of the experiment to match.

Development • Supplementary information

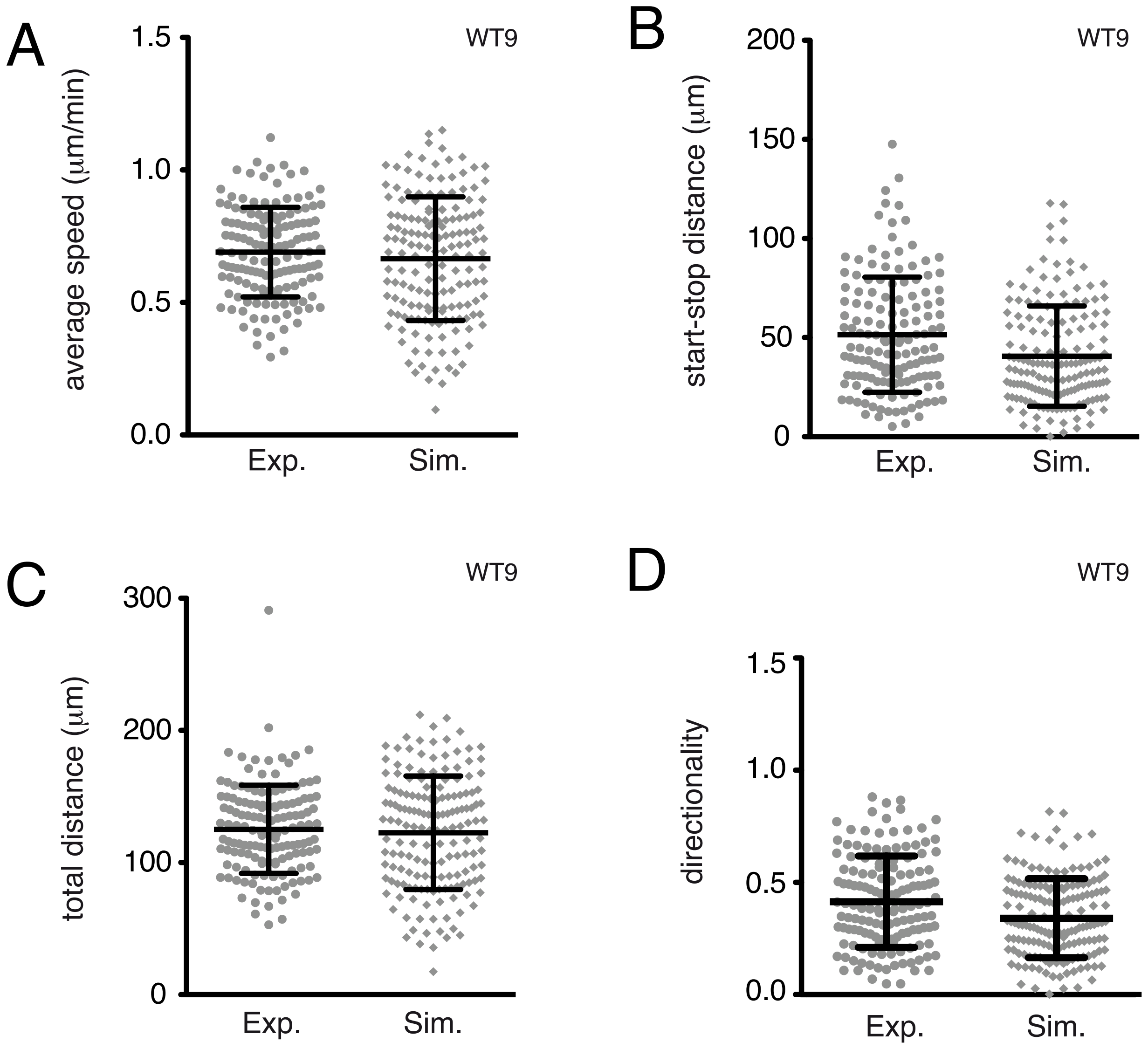


**Figure S1. Geometric characteristics of cell trajectories associated with distances and extracted by experimental movies and mathematical model.**

A. Average speed of Mb extracted from the experimental movies WT9 (Exp.) and the mathematical model (Sim.) from the retrieved sets of coordinates.

B. Euclidian distance of Mb trajectories extracted from the experimental movies WT9 (Exp.) and the mathematical model (Sim.) from the retrieved sets of coordinates.

C. Total distance of Mb trajectories extracted from the experimental movies WT9 (Exp.) and the mathematical model (Sim.) from the retrieved sets of coordinates.

D. Directionality of Mb trajectories extracted from the experimental movies WT9 (Exp.) and the mathematical model (Sim.) from the retrieved sets of coordinates.

Statistics were performed using the non-parametric Mann-Whitney t-test. (A) p-value = 0.3752, (B) p-value = 0.0006, (C) p-value = 0.7803, (D) p-value = 0.002.

Development • Supplementary information

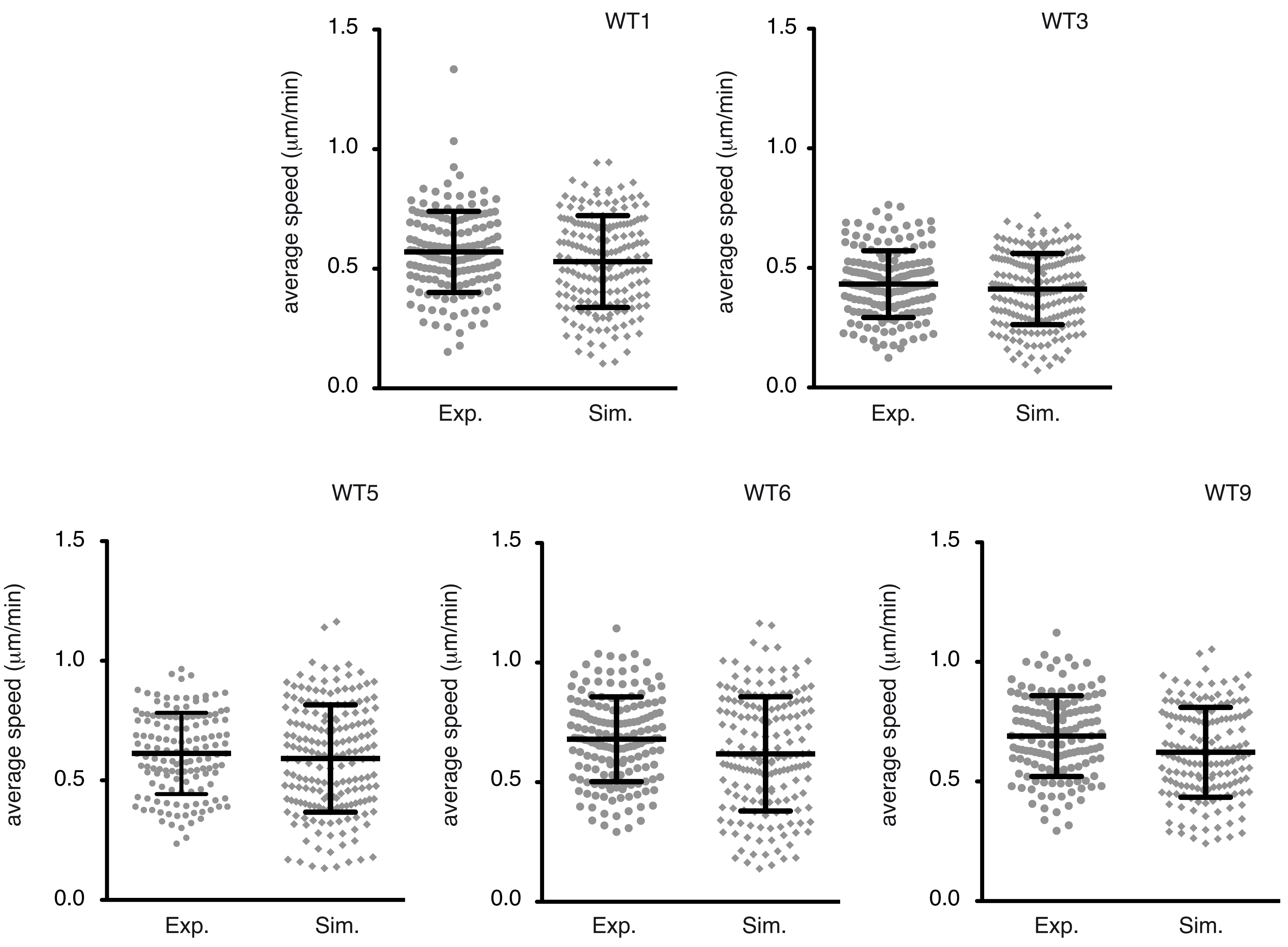


**Figure S2. Average speed of melanoblasts extracted from experimental movies and mathematical model.**

Average speed of Mb extracted from the experimental movies (Exp.) and the mathematical model (Sim.) from the retrieved sets of coordinates of five independent experiments (WT1, WT3, WT5, WT6 and WT9). For each movie from about 140 to 220 Mb were followed. For the simulations, an equivalent number of Mb was challenged. Statistics were performed using the non-parametric Mann-Whitney t-test. (WT1) p-value = 0.1059, (WT3) p-value =0.3651, (WT5) p-value = 0.3984, (WT6) p-value = 0.0178, (WT9) p-value = 0.0026.

Development • Supplementary information

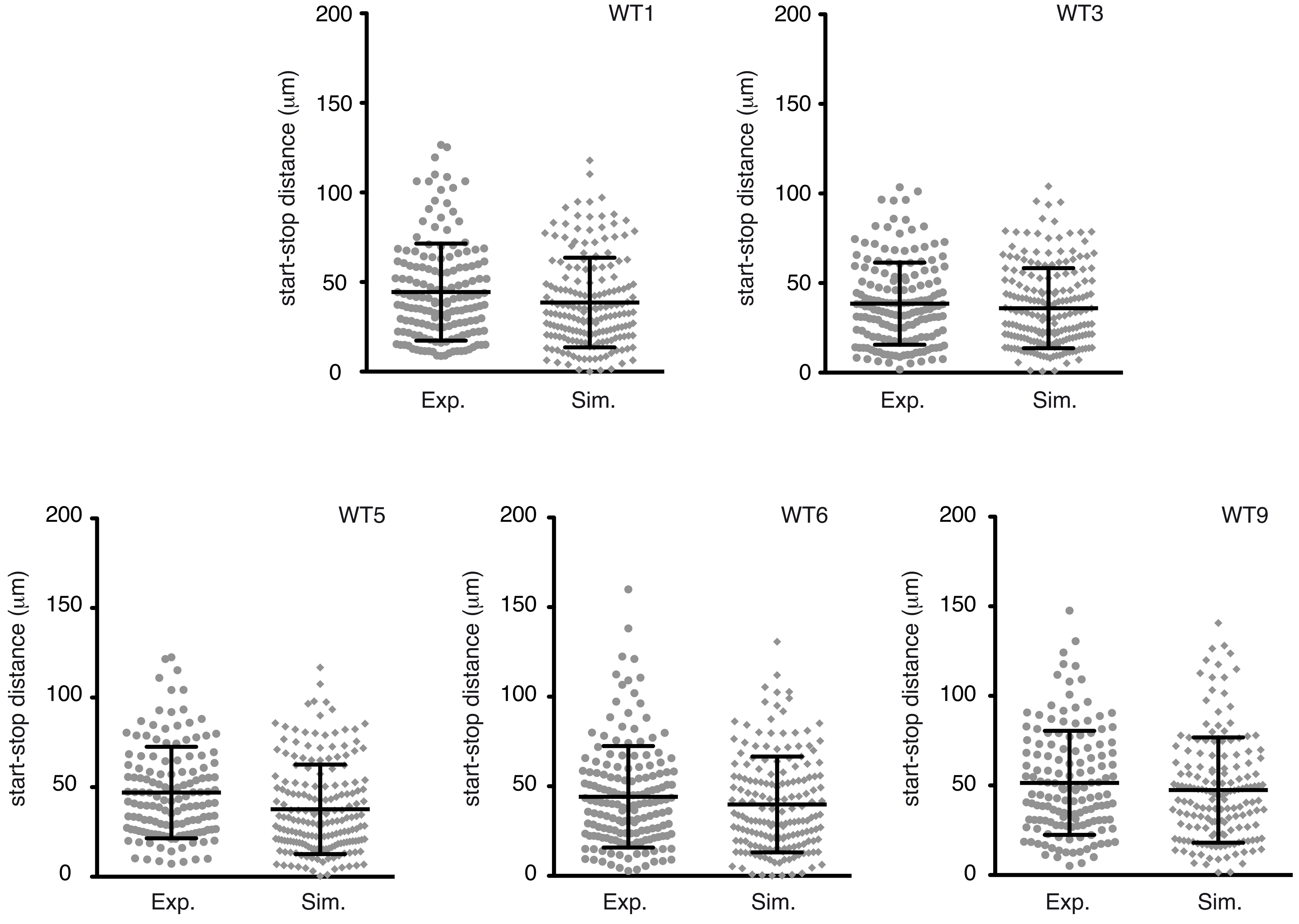


**Figure S3. Euclidian distances of melanoblasts trajectories extracted from experimental movies and mathematical model.**

Euclidian distance of Mb trajectories extracted from the experimental movies (Exp.) and the mathematical model (Sim.) from the retrieved sets of coordinates of five independent experiments (WT1, WT3, WT5, WT6 and WT9). For each movie from about 140 to 220 Mb were followed. For the simulations, an equivalent number of Mb was challenged. Statistics were performed using the non-parametric Mann-Whitney t-test. (WT1) p-value = 0.0476, (WT3) p-value =0.3056, (WT5) p-value = 0.0002, (WT6) p-value = 0.1554, (WT9) p-value = 0.1827.

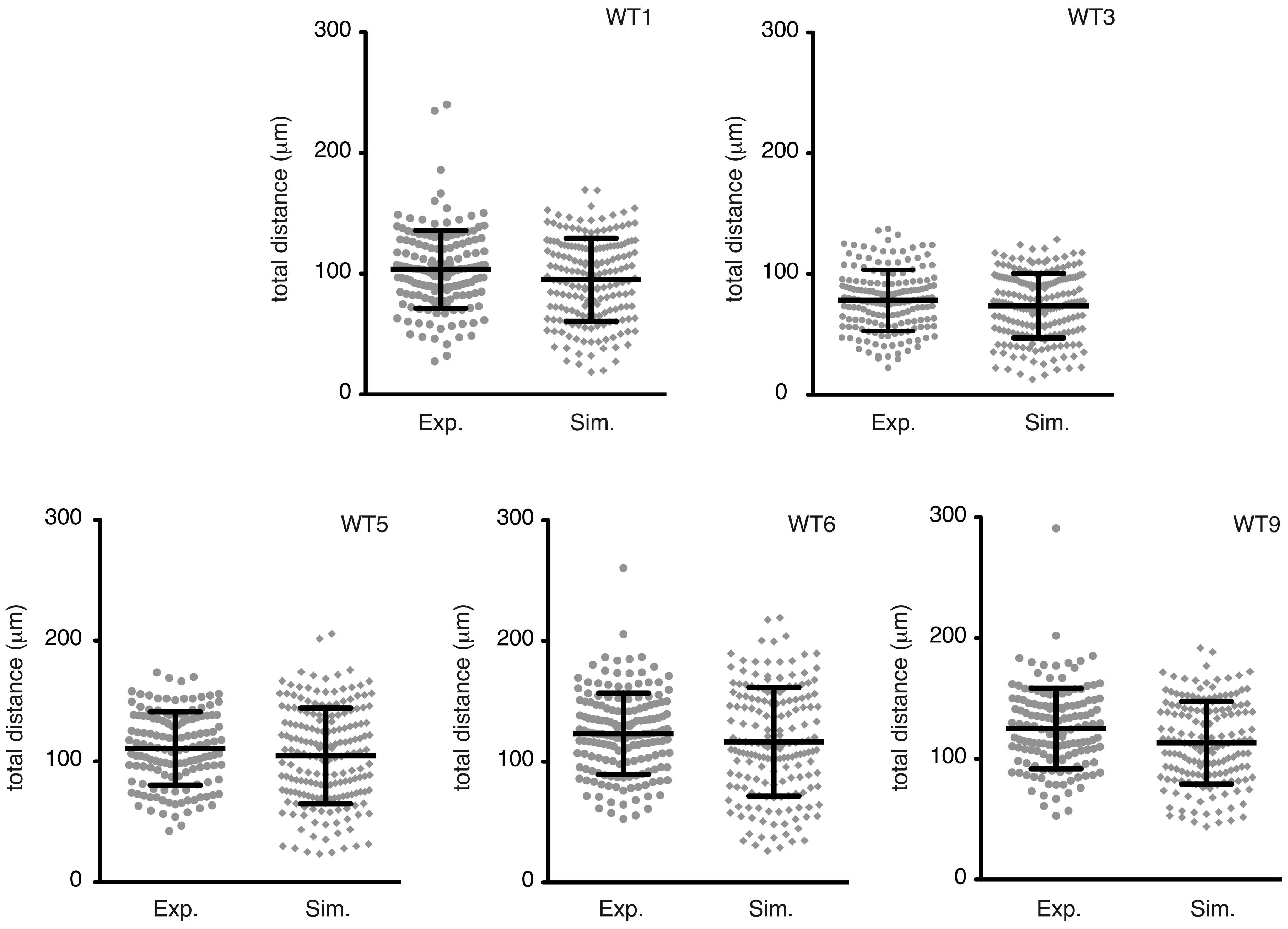


**Figure S4. Total distances of melanoblasts trajectories extracted from experimental movies and mathematical model.**

Total distance of Mb trajectories extracted from the experimental movies (Exp.) and the mathematical model (Sim.) from the retrieved sets of coordinates of five independent experiments (WT1, WT3, WT5, WT6 and WT9). For each movie from about 140 to 220 Mb were followed. For the simulations, an equivalent number of Mb was challenged. Statistics were performed using the non-parametric Mann-Whitney t-test. (WT1) p-value = 0.0720, (WT3) p-value =0.2384, (WT5) p-value = 0.1722, (WT6) p-value = 0.1820, (WT9) p-value = 0.0070.

Development • Supplementary information

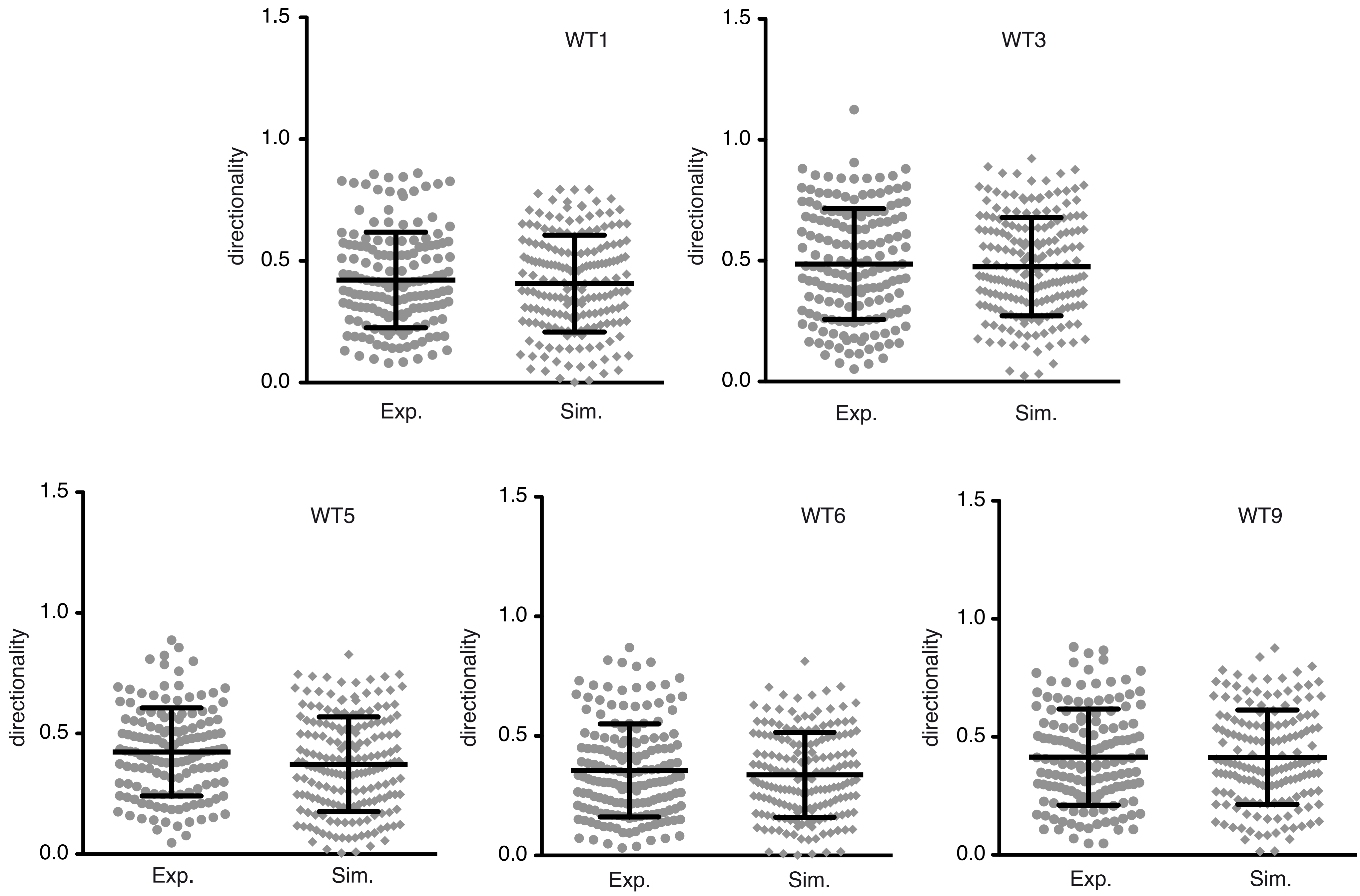


**Figure S5. Directionality of melanoblasts extracted from experimental movies and mathematical model.**

Directionality of Mb trajectories extracted from the experimental movies (Exp.) and the mathematical model (Sim.) from the retrieved sets of coordinates of five independent experiments (WT1, WT3, WT5, WT6 and WT9). For each movie from about 140 to 220 Mb were followed. For the simulations, an equivalent number of Mb was challenged. Statistics were performed using the non-parametric Mann-Whitney t-test. (WT1) p-value = 0.7534, (WT3) p-value =0.6558, (WT5) p-value = 0.0245, (WT6) p-value = 0.6691, (WT9) p-value = 0.8662.

Development • Supplementary information

**List of values**

| | |
|---|---|
| Number of Kc | 4000 |
| Number of Mb | ≈ 220 |
| Average Kc size (µm) | 10 |

**List of parameters**

| | | |
|---|---|---|
| Attraction potential of Kc for Mb | Cf : Increase of the attraction potential of Kc for Mb<br>Cb : Decrease of the attraction potential of Kc for Mb.<br>$(C_b = 10C_f)$ | 0.2 |
| Diffusion of Kc potential | $v_d$ : parameter of diffusion of Kc potential | $0 \leq v_d \leq 1$ |
| Repulsion between Mb | ρ : Parameter of repulsion<br>Me: Parameter of repulsion | ρ = ~0.8 |

**Table S1**. Values and parameters used in the mathematical model

Development • Supplementary information

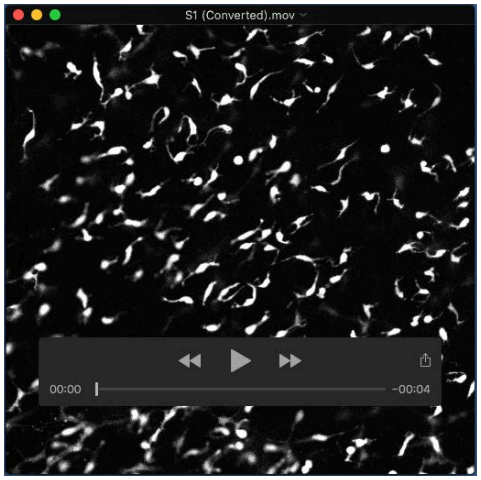


Movie S1. The movie is created from WT1 consisting of 38 images that were taken every five minutes. The resulting images of 512 × 512 pixels, representing a square with sides of 640µm, show the melanoblasts, made visible by auto-fluorescence, on the basal membrane of the epidermis.

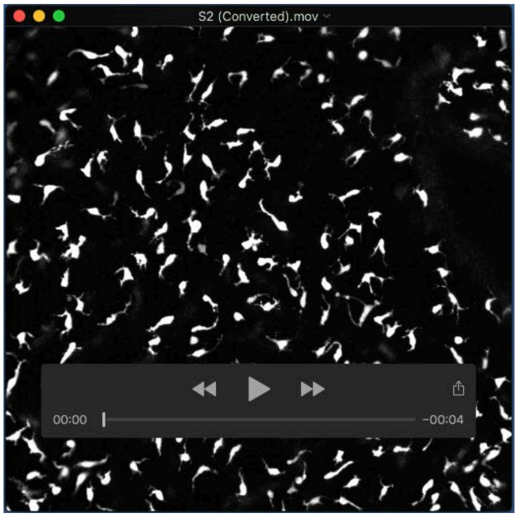


Movie S2.The movie is created from WT2 consisting of 38 images that were taken every five minutes. The resulting images of 512 × 512 pixels, representing a square with sides of 640µm, show the melanoblasts, made visible by auto-fluorescence, on the basal membrane of the epidermis.

Development • Supplementary information

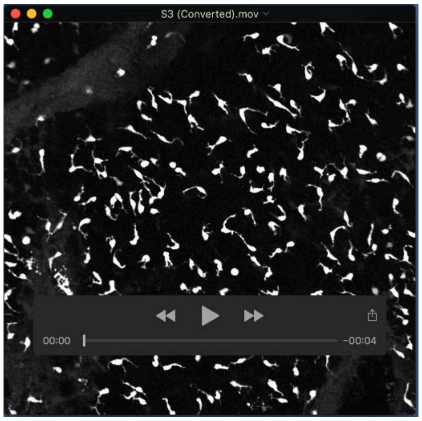


Movie S3. The movie is created from WT3 consisting of 38 images that were taken every five minutes. The resulting images of 512 × 512 pixels, representing a square with sides of 640µm, show the melanoblasts, made visible by auto-fluorescence, on the basal membrane of the epidermis.

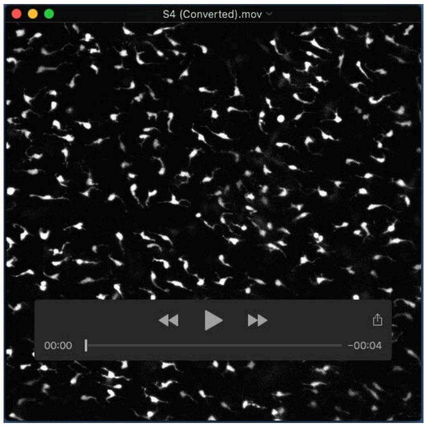


Movie S4. The movie is created from WT4 consisting of 38 images that were taken every five minutes. The resulting images of 512 × 512 pixels, representing a square with sides of 640µm, show the melanoblasts, made visible by auto-fluorescence, on the basal membrane of the epidermis.

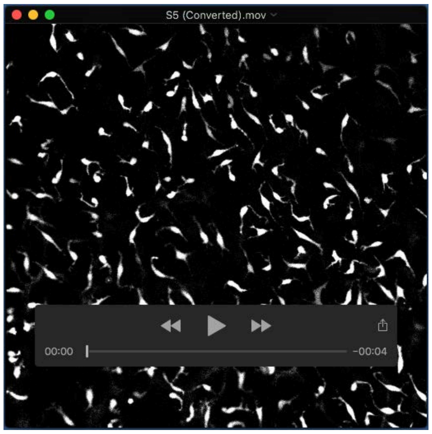


Movie S5. The movie is created from WT5 consisting of 38 images that were taken every five minutes. The resulting images of 512 × 512 pixels, representing a square with sides of 640µm, show the melanoblasts, made visible by auto-fluorescence, on the basal membrane of the epidermis.

Development • Supplementary information

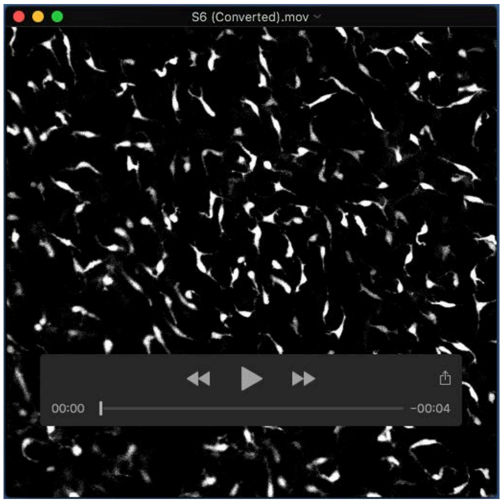


Movie S6. The movie is created from WT6 consisting of 38 images that were taken every five minutes. The resulting images of 512 × 512 pixels, representing a square with sides of 640µm, show the melanoblasts, made visible by auto-fluorescence, on the basal membrane of the epidermis.

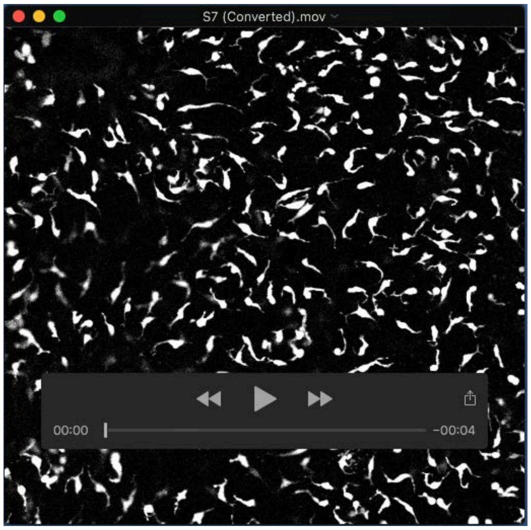


Movie S7. The movie is created from WT7 consisting of 38 images that were taken every five minutes. The resulting images of 512 × 512 pixels, representing a square with sides of 640µm, show the melanoblasts, made visible by auto-fluorescence, on the basal membrane of the epidermis.

Development • Supplementary information

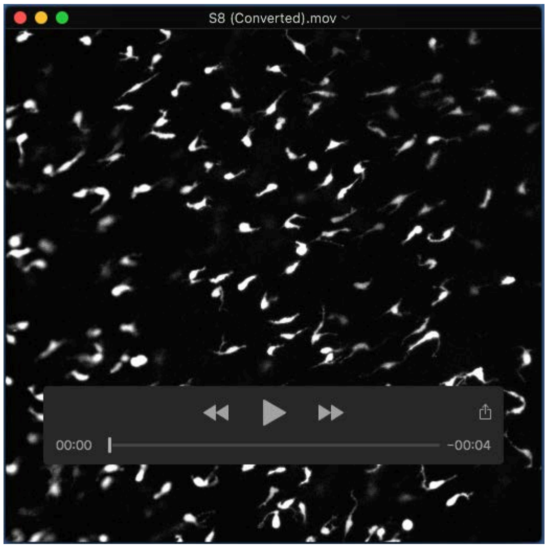


Movie S8. The movie is created from WT8 consisting of 38 images that were taken every five minutes. The resulting images of 512 × 512 pixels, representing a square with sides of 640µm, show the melanoblasts, made visible by auto-fluorescence, on the basal membrane of the epidermis.

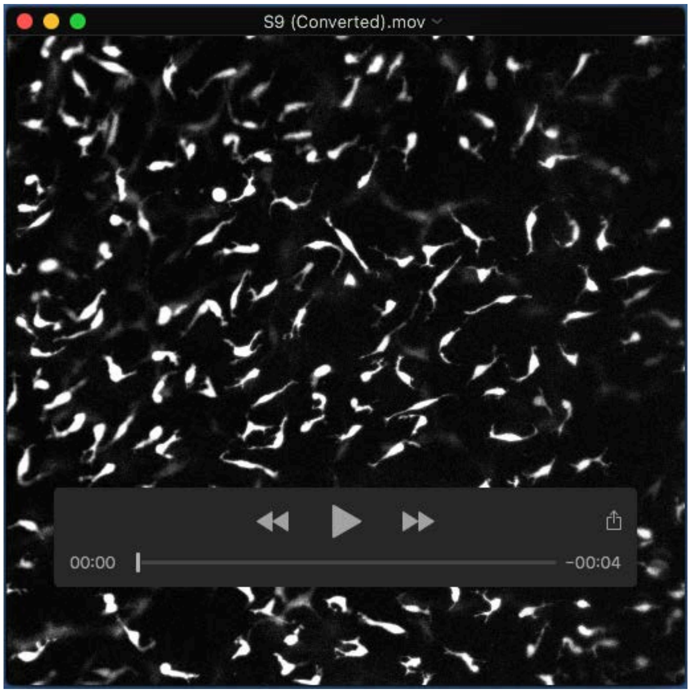


Movie S9. The movie is created from WT9 consisting of 38 images that were taken every five minutes. The resulting images of 512 × 512 pixels, representing a square with sides of 640µm, show the melanoblasts, made visible by auto-fluorescence, on the basal membrane of the epidermis._
